# Chronic SSRI Treatment, but Not Norepinephrine Reuptake Inhibitor Treatment, Increases Neurogenesis in Juvenile Rats

**DOI:** 10.3390/ijms23136919

**Published:** 2022-06-22

**Authors:** Michelle Hovorka, David Ewing, David S. Middlemas

**Affiliations:** Kirksville College of Osteopathic Medicine, A.T. Still University, Kirksville, MO 63501, USA; mhorvorka@atsu.edu (M.H.); dewing@atsu.edu (D.E.)

**Keywords:** antidepressant drugs, adolescent, major depressive disorder, neurogenesis

## Abstract

There has been growing recognition that major depressive disorder is a serious medical disorder that also affects children. This has been accompanied by an increased use of antidepressant drugs in adolescents; however, not all classes of antidepressants are effective in children and adolescents. There is an increasing need to understand the differences in antidepressant action in different developmental stages. There are some data indicating that the behavioral effect of chronic antidepressant treatment in adult rodents is dependent on hippocampal neurogenesis; however, it is not known which classes of antidepressant drugs induce hippocampal neurogenesis in adolescent rodents. Three classes of antidepressant drugs were tested in two age groups of Sprague Dawley rats, pre-adolescent (postnatal days 11–24) and adolescent (postnatal days 21–34): monoamine oxidase inhibitors (MAOIs); selective serotonin reuptake inhibitors (SSRIs); serotonin norepinephrine reuptake inhibitors (SNRIs); and tricyclic antidepressants (TCAs). To address which classes of antidepressant drugs might alter the rate of mitogenesis in neural progenitor cells in an adolescent rodent model, adolescent Sprague Dawley rats were treated with the thymidine analog 5-bromo-deoxy-2′-uridine (BrdU) on postnatal days 21 and 22 and antidepressant drugs or vehicle for 14 days (postnatal days 21–34). To address which classes of antidepressant drugs might alter the rate of neurogenesis, postnatal day-21 Sprague Dawley rats were treated with antidepressant drugs or vehicle for 14 days (postnatal days 21–34) and BrdU on postnatal days 33 and 34. In both experimental paradigms, BrdU-positive cells in the subgranular zone and the granule cell layer were counted. Newborn neurons were identified in the neurogenic paradigm by identifying cells expressing both the neuronal specific marker NeuN and BrdU using confocal microscopy. Only the SSRI fluoxetine significantly altered the basal mitogenic and neurogenic rates in adolescent rats. Treatment with the monoamine oxidase inhibitor (MAOI) tranylcypromine (TCP) and the TCA desipramine did not alter the rate of hippocampal neurogenesis in the adolescent rats. This is consistent with human clinical observations, where only SSRIs have efficacy for treatment of depression in patients under the age of 18. In pre-adolescent rats, postnatal days 11–24, none of the drugs tested significantly altered the basal mitogenic or neurogenic rates. All of the classes of antidepressant drugs are known to induce hippocampal neurogenesis in adult rats. The mechanisms of action underlying this developmental difference in antidepressant drug action between juveniles and adults are not known.

## 1. Introduction

There are developmental differences in the responses to antidepressant drugs. Children respond differently than adults to antidepressant drugs. Children respond to SSRIs [1,2]. Children do not respond to most tricyclic antidepressants [3,4], which inhibit the reuptake of norepinephrine, serotonin, and dopamine with varying efficacy. Tricyclic antidepressants vary in which transmitter reuptake they inhibit the most. The reason for these developmental differences in antidepressant drug class efficacy is not understood. Guidelines definitively call for the use of antidepressant drugs, specifically with an SSRI or psychotherapy or both in adolescents [5]. Only two drugs are approved by the FDA for use in adolescents for depression, fluoxetine and escitalopram. Both are SSRIs. The reason for this underlying difference in antidepressant drug class efficacy between adolescents and adults is not known, but it can be postulated that it is caused by underlying differences in the developing nervous system. The serotonergic neurotransmitter systems mature earlier during development than the adrenergic neurotransmitter systems. This earlier maturation of the serotonergic system may explain why SSRIs are effective in adolescents, whereas noradrenergic drugs lack efficacy. Notably, the development of the human nervous system spans two decades or more. Regardless, there is a clear need to understand the developmental differences in antidepressant drug action between adolescents and adults.

**Figure 1:**
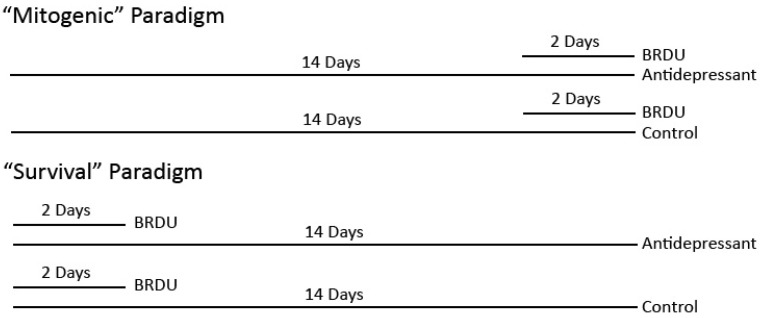
Experimental paradigms. The “survival” paradigm we developed calls for coadministration of BrdU for two days at the beginning of a 14-day treatment regimen with or without antidepressant drug, whereas the “mitogenic” paradigm calls for coadministration of BrdU for two days at the end of the 14-day treatment. Preliminary experiments indicated two days of BrdU administration would allow optimal labeling for our experimental design (data not shown). Two developmental stages of Sprague Dawley rats were tested: pre-adolescent (11–24 days) and adolescent (21–34 days).

Antidepressant drugs induce hippocampal neurogenesis in the adult rat hippocampus [6]. Neurogenesis is required for some antidepressant drug actions in adult rodents. Radiation, which blocks neurogenesis, was also observed to blocked some antidepressant drug action [7]. Behavioral studies have found that escitalopram and fluoxetine (SSRIs), but not a noradrenergic selective reuptake inhibitor, have antidepressant-like effects in juvenile rats [8]. Moreover, escitalopram, but not desipramine, induces brain-derived neurotrophic factor (BDNF) expression in the hippocampus [9,10]. This study tests whether three different classes of antidepressant drugs induce neurogenesis in juvenile rats. Desipramine preferentially inhibits norepinephrine reuptake, whereas fluoxetine selectively inhibits serotonin reuptake. Tranylcypromine inhibits monoamine oxidase, which is required for the degradation of norepinephrine, serotonin, and dopamine. Specifically, this study tests whether different classes of antidepressant drugs alter hippocampal adult neurogenesis in the dorsal hippocampus in two age groups of juvenile rats using the experimental paradigms in Figure 1.

We used two experimental paradigms to test our hypotheses (Figure 1). (1) We tested whether antidepressant drugs alter the basal mitogenic rate of neural progenitor cells in the hippocampus, and (2) we tested whether antidepressant drugs alter neurogenesis in the dentate gyrus. Moreover, we tested this at two distinct developmental stages in juvenile Sprague Dawley rats: pre-adolescent, postnatal days 11–24 (PND 11–24), and adolescent, postnatal days 21–34 (PND 21–34). The goal was to test pre-pubescent rats and rats spanning puberty. It is anticipated the results will allow us to determine whether there are developmental differences in the mitogenic and neurogenic effects of antidepressant drugs and whether there are antidepressant drug class differences in juvenile rats. We used two different paradigms to address whether antidepressant drugs alter the basal mitogenic rate of neural precursor cells or neurogenesis (Figure 1). (1) In the first “mitogenic” paradigm, rat pups were treated with or without an antidepressant drug, and BrdU was co-administered at the end of the treatment schedule. (2) In the second “survival” paradigm, rat pups were treated with or without an antidepressant drug, and BrdU was co-administered at the beginning of the treatment schedule.

## 2. Results

**Figure 2:**
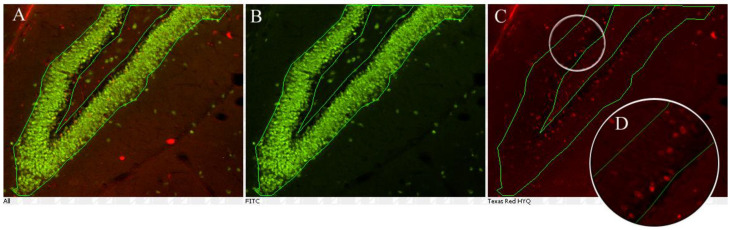
Counting BrdU-positive cells in the granule cell layer and about two cell diameters of the SGZ. The apex of the adolescent rat dentate gyrus showing NeuN (green) and BrdU (red) immunoreactivity is imaged. (**A**) The dentate gyrus and subgranular zone outlined in the overlayed image. (**B**) NeuN immunoreactivity. (**C**) BrdU immunoreactivity. (**D**) A magnified portion of the granule cell layer showing BrdU immunoreactivity used for cell counting.

**Figure 3:**
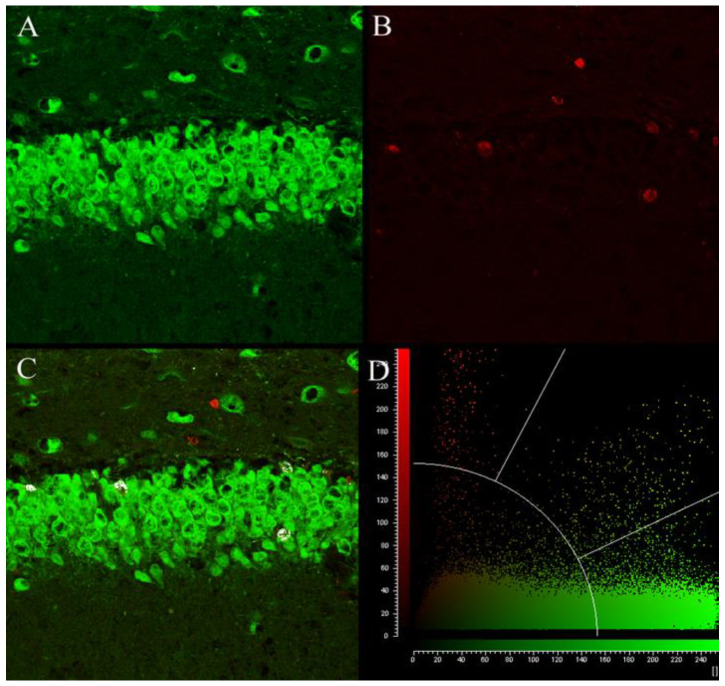
Determination of colocalization of fluorescence using confocal microscopy. Confocal microscopy was used to determine colocalization of the signal derived from immunohistochemical detection of BrdU and NeuN. Confocal images of the granule cell layer and subgranular zone of the dentate gyrus double-labeled for NeuN (green) and BrdU (red) with Alexa 488 and Alexa 594 fluorescent dyes, respectively, are shown. (**A**) NeuN immunoreactivity. (**B**) BrdU immunoreactivity. (**C**) Overlayed image of NeuN and BrdU immunoreactivity with colocalized pixels highlighted in white. (**D**) A graph of pixel intensities and color of the overlayed image. The pixels of low-intensity signal below the semi-circle are considered background. Pixels to the left and right of the signal threshold lines are considered separate, while pixels with high green and red signal intensity in the upper right quadrant of the graph are considered colocalized. Colocalized pixels are highlighted in white in panel (**C**).

We determined whether chronic antidepressant drug treatment (14 days) changed the number of newborn cells in the “survival” paradigm in pre-adolescent (days 11–24) and adolescent (days 21–34) rats (Figure 1). In this paradigm, newborn cells are marked with BrdU in the first 2 days of treatment. A quantitative assay for mitogenesis was developed using BrdU labeling and fluorescent immunohistochemical detection for counting newborn cells (Figure 2), and a quantitative neurogenesis assay was developed using colocalization of the fluorescent signal arising from BrdU and NeuN using confocal microscopy (Figure 3).

**Figure 4:**
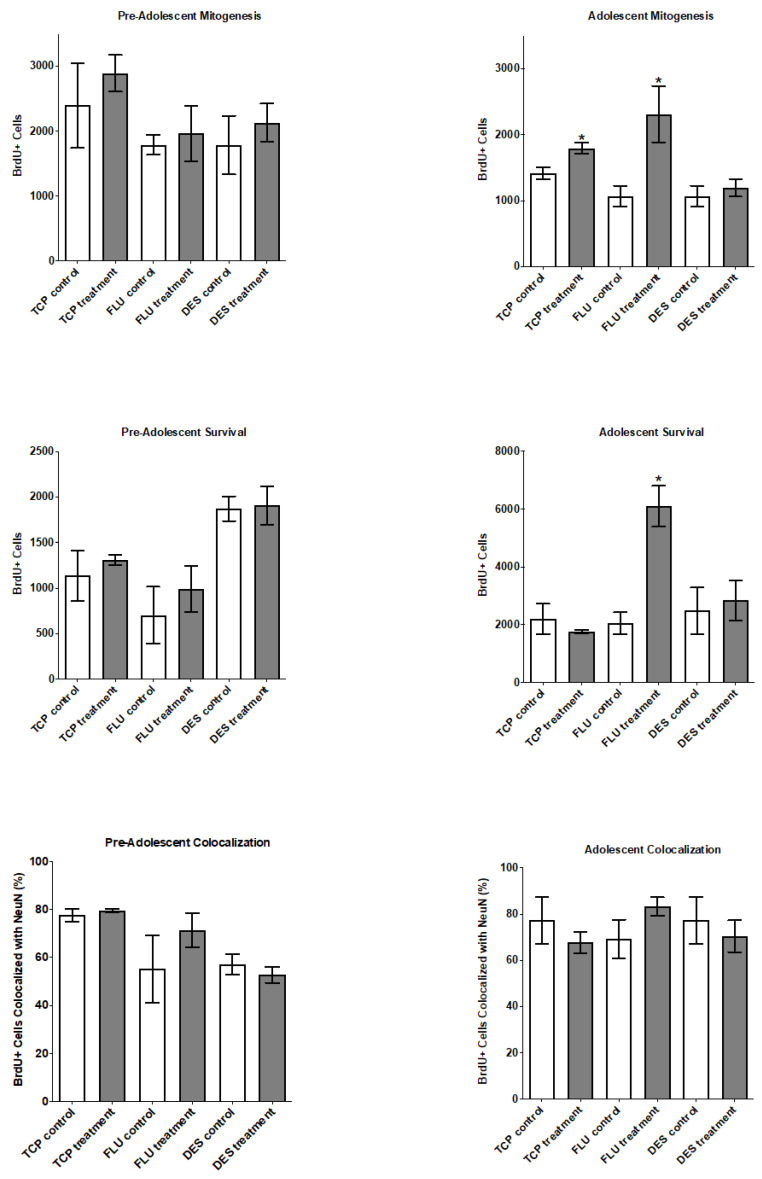
Effect of TCP, fluoxetine, and desipramine on birth (upper panels) or survival (middle panels) of newborn cells in the juvenile rat hippocampus in pre-adolescent (days 11–24) and adolescent (days 21–34) rats. Upper panels: Effect of TCP, fluoxetine, and desipramine on birth of newborn cells in the juvenile rat hippocampus in pre-adolescent (day 11–24) and adolescent (day 21–34) rats. The total number of BrdU-positive cells in the granule cell layer after 14 days of treatment with TCP (10 mg∙kg^−1^∙day^−1^) or vehicle, fluoxetine (4 mg∙kg^−1^∙day^−1^) or vehicle, and desipramine (7 mg∙kg^−1^∙day^−1^). BrdU (25 mg∙kg^−1^∙day^−1^) was administered twice daily for the last two days of treatment in the “mitotic” paradigm. Values are expressed as means ± SE. Statistical analysis with an unpaired, one-tailed *t*-test demonstrates that the results are not significant based on a definition of * *p* < 0.05 (*n* = 4 in all groups except *n* = 5 in the adolescent desipramine group). Middle panels: Effect of TCP, fluoxetine, and desipramine on survival of newborn cells in the juvenile rat hippocampus in pre-adolescent (days 11–24) and adolescent (days 21–34) rats. The total number of BrdU-positive cells in the granule cell layer after 14 days of treatment with TCP (10 mg∙kg^−1^∙day^−1^) or vehicle, fluoxetine (4 mg∙kg^−1^∙day^−1^) or vehicle, and desipramine (7 mg∙kg^−1^∙day^−1^). BrdU (25 mg∙kg^−1^∙day^−1^) was administered twice daily for the first two days of treatment in the “survival” paradigm. Values are expressed as means ± SE. Statistical analysis with an unpaired, one-tailed *t*-test demonstrated that the results are not significant based on a definition of * *p* < 0.05 (*n* = 4 in all groups except *n* = 5 in the adolescent desipramine group, *n* = 6 in the adolescent TCP group). Lower panels: Effect of TCP, fluoxetine, and desipramine on differentiation. Depiction of BrdU-positive cells in the granule cell layer that are colocalized with NeuN divided by the total number of BrdU-positive cells (%). The data are derived from the same samples used in the upper panels. Values are expressed as means ± SE. Statistical analysis with an unpaired, one-tailed *t*-test demonstrated that the results are not significant based on a definition of *p* < 0.05.

We determined whether chronic antidepressant drug treatment (14 days) changed the number of newborn cells in the “mitogenic” paradigm in pre-adolescent (days 11–24) and adolescent (days 21–34) rats. In this paradigm, newborn cells are marked with BrdU after 14 days of treatment. Tranylcypromine and desipramine dosing was based upon functional response in juvenile rats using the forced swim test [8,10]. Fluoxetine dosing was based upon functional response in the force swim test [8] and other behavioral responses [11] in juvenile rats. The antidepressant drugs from the three different classes did not significantly alter the birth of newborn cells after 14 days of treatment in pre-adolescent (days 11–24) rats (Figure 4, upper left panel). Very notably, fluoxetine did induce a significant increase in the number of newborn cells, i.e., increase the mitogenic rate, after 14 days of treatment in adolescent (days 21–34) rats (Figure 4, upper right panel). To a lesser extent, TCP induced a significant increase in the number of newborn, i.e., increase the mitogenic rate, after 14 days of treatment in adolescent (days 21–34) rats (Figure 4, upper right panel).

The antidepressant drugs from the three different classes did not significantly alter the number of newborn cells “surviving” after 14 days of treatment in pre-adolescent (days 11–24) rats (Figure 4, middle left panel). Notably, fluoxetine did induce an increase in the number of newborn cells “surviving” after 14 days of treatment in adolescent (days 21–34) rats (Figure 4, middle right panel).

Moreover, the ratio of newborn neurons to total newborn cells was not altered by any of the drugs (Figure 4, lower panels). Since the ratio of cells becoming new neurons is not changed by antidepressant drug treatment, it suggests antidepressant drugs also increase gliogenesis in the adolescent rats.

**Figure 5:**
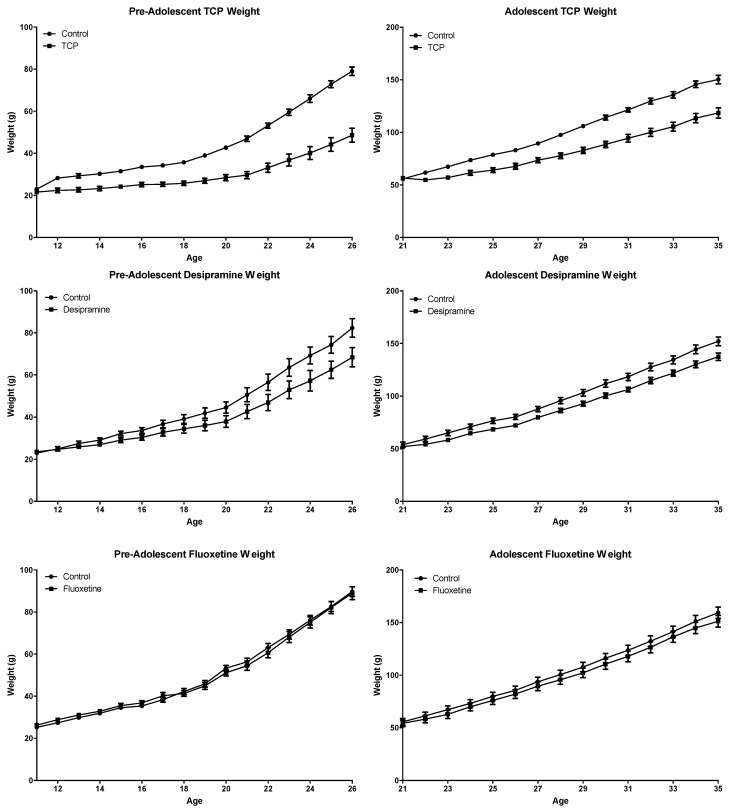
Growth of pre-adolescent (days 11–24) and adolescent (days 21–34) rats with chronic antidepressant treatment. TCP (10 mg∙kg^−1^∙day^−1^) or vehicle, fluoxetine (4 mg∙kg^−1^∙day^−1^) or vehicle, and desipramine (7 mg∙kg^−1^∙day^−1^) were administered once daily i.p. for 14 days. Values are expressed as means ± SE. Statistical analysis was performed with a two-way ANOVA based on a definition of *p* < 0.05 as significant. The growth curves were significantly different for pre-adolescent TCP (DF = 1, f = 8, *p* < 0.0001), adolescent TCP (DF = 1, f = 373, *p* < 0.0001), pre-adolescent desipramine (DF = 1, f = 37, *p* < 0.0001), adolescent desipramine (DF = 1, f = 87, *p* < 0.0001), and adolescent fluoxetine (DF = 1, f = 8, *p* = <0.005). The growth curves were not significantly different for pre-adolescent fluoxetine (DF = 1, f = 0.09, *p* = 0.76). The interaction between age and treatment was significant for TCP- and desipramine-treated rats (*p* < 0.0001) but was not significant for fluoxetine-treated rats (*p* > 0.05).

Chronic administration (14 days) of TCP, and to a lesser extent desipramine, significantly decreased the growth rate of both pre-adolescent (days 11–24) and adolescent (days 21–34) rats (Figure 5). Fluoxetine slightly altered the growth rate of adolescent rats but did not significantly alter the growth rate of pre-adolescent rats. The results are intriguing and suggest a noradrenergic, or perhaps dopaminergic, rather than a serotonergic, mechanism is involved in the differing growth rates.

## 3. Discussion

The mitogenic and survival-enhancing effects of fluoxetine in adolescent rats are consistent with the ability of SSRIs to relieve depression in minors [12]. The reason that rates of mitogenesis and neuron survival did not change in pre-adolescent rats in response to fluoxetine is not known. However, one hypothesis is that the higher rate of mitogenesis in pre-adolescent rats masks any effect of the antidepressant. It is important to address the mechanism of action in juvenile models, since SSRIs are increasingly used to treat pediatric depression despite controversy over increased suicidal ideation. Indeed, the benefits of antidepressants appear to be much greater than risks from suicidal ideation and suicide attempt in adolescents [13].

Corticosterone increases depressive-like behavior in adult rats [14]. It is striking that in pre-pubescent rats, chronic corticosterone treatment did not affect behavior or neurogenesis in female pre-pubescent juvenile rats [15]. However, in post-pubescent rats, chronic corticosterone showed increased depressive-like behavior, as well as a decrease in cell proliferation in the subgranular zone [15]. In both, a similar but inverse result resembles the findings here in that antidepressant drugs have little effect on neurogenesis in pre-adolescent rats, but SSRIs increase neurogenesis in adolescent rats.

The increase in neurogenesis in adolescent rats in response to chronic SSRI treatment also parallels the increase in hippocampal neurogenesis seen in studies of the effect of all classes of antidepressants on adult rats [7,16,17,18]. During development, the serotonergic system develops early, while the adrenergic system does not fully develop until early adulthood [19]. The inability of desipramine (NE reuptake inhibitor) to significantly alter the rate of mitogenesis or survival in either the adolescent juvenile or adolescent age group correlates with later development of the noradrenergic systems relative to serotonergic systems. Interestingly, tranylcypromine (MAOI) increased mitogenesis but not neurogenesis in adolescent rats, perhaps due to the inhibition of serotonin catabolism.

Brain-derived neurotrophic factor (BDNF) and its receptor TrkB [20,21] may have a role in the mechanism of fluoxetine on neurogenesis and survival. Treatment with escitalopram, an SSRI, has been shown to increase the level of BDNF and TrkB proteins in juvenile rats, while norepinephrine reuptake inhibitors do not alter the level of BDNF or TrkB proteins in rats of the same age [9,10]. It is indeed striking that the data in this report on an increase in neurogenesis induced by an SSRI, but not a norepinephrine-selective reuptake inhibitor, correlate well with observations that only SSRIs induce BDNF expression in juvenile rats. This indeed suggests that BDNF and neurogenesis are involved in antidepressant action and, moreover, may underlie the developmental differences in antidepressant drug class action observed in patients.

A profound decrease in growth rate was observed in both pre-adolescent (days 11–24) and adolescent (days 21–34) rats treated with TCP, while a modest decrease was observed following desipramine treatment. There is, however, not a significant difference in the growth rate in either age group of juvenile rats treated with fluoxetine. We observed this in both rats feeding primarily from a mother and rats feeding independently. We do not know whether the underlying mechanism for this observation of the effect of TCP and desipramine is behavioral (appetite or exercise) or metabolic. The decreased growth rate seems to be correlated with a noradrenergic or dopaminergic mechanism rather than a serotonergic mechanism.

## 4. Materials and Methods

### 4.1. Drug Treatment

For the 11–24-day cohorts, eight 11-day-old male Sprague Dawley rats were randomly divided into two groups (*n* = 4), housed with one mother (Hilltop Laboratories), and treated for 14 days with or without an antidepressant drug twice daily i.p. A group number of four was selected based on previous work [10]. Tranylcypromine (10 mg∙kg^−1^∙day^−1^), desipramine (7 mg∙kg^−1^∙day^−1^), or fluoxetine (4 mg∙kg^−1^∙day^−1^) was used for both experimental paradigms. BrdU (25 mg∙kg^−1^∙day^−1^) was coadministered for two days twice daily i.p. at the beginning or the end of the regimen, depending on which experimental paradigm was being tested.

For the 21–34-day cohorts, eight 21-day-old male Sprague Dawley rats were randomly divided into two groups (*n* = 4) and housed with four rats per cage (Hilltop Laboratories). Two saline control rats and two drug-treated rats were housed per cage. The rats were then treated for 14 days with or without antidepressant drugs, and BrdU (25 mg∙kg^−1^∙day^−1^) was co-administered for two days twice daily i.p. at the beginning or end of the regimen, depending on which experimental paradigm was being tested. Tranylcypromine (10 mg∙kg^−1^∙day^−1^), desipramine (7 mg∙kg^−1^∙day^−1^), or fluoxetine (4 mg∙kg^−1^∙day^−1^) was used for both experimental paradigms.

Brains were harvested on the 15th day of treatment (PND 25 for pre-adolescent rats and PND 35 for adolescent rats) from anesthetized (sodium pentobarbital) rat pups using transcardial perfusion with 30 mL normal saline followed by 60 mL 4% paraformaldehyde. Brains were placed in 30% sucrose for 3 days and frozen at −80 °C. The specimens were coronally sectioned on a Leica CMI900 cryostat at −22 °C. Every eighth 30 μm section from the equivalent of 3.14 to 4.52 mm posterior to the bregma in adult brain [22] was collected and stored in phosphate-buffered saline solution with 0.01% sodium azide preservative. Slicing was started at the equivalent of plate 32 of adult rat brain [22] and encompassed the juvenile dorsal hippocampus. The BrdU-positive cells were quantified using a semi-stereological protocol described previously [18,23].

### 4.2. Immunohistochemical Assay for Mitogenesis in the Dorsal Hippocampus

A quantitative assay for mitogenesis (Figure 2) was developed using BrdU labeling and fluorescent immunohistochemical detection for counting newborn cells. Slices were analyzed using fluorescent immunohistochemistry. Immunohistochemistry staining was performed on free-floating brain sections and washed with 0.1 M phosphate-buffered saline (PBS). Sections were first incubated in 1 M HCl for 1 h at 37 °C to denature the DNA in preparation for the BrdU primary antibody. All sections were then pre-treated with 3% bovine serum albumin in 0.1 M PBS, 0.15% Triton X-100 for 1 h at 22 °C. The primary antibodies used were rat anti-BrdU (1:4000; AbD Serotec, Raleigh, NC, USA) and mouse anti-NeuN (1:4000; Millipore Corp., Chicago, IL, USA). Sections were incubated overnight at 4 °C with the primary antibodies diluted in 1% BSA in 0.1 M PBS, 0.15% Triton X-100. Thereafter, brain slices were incubated for 2 h at RT with the secondary antibodies anti-rat conjugated with Alexa fluor 594 (1:1000; Invitrogen, Chicago, IL, USA) and anti-mouse conjugated with Alexa fluor 488 (1:1000; Invitrogen) in 0.2% BSA in 0.1 M PBS, 0.15% Triton X-100. Sections were mounted on pre-treated slides (Superfrost Plus; Fisher Scientific, Pittsburgh, PA, USA). Coverslips were attached using tissue mount (ProLong Gold; Invitrogen, Waltham, MA, USA) and dried overnight.

Fluorescence was detected using epifluorescence microscopy (Nikon ECLIPSE 80i, Nikpon Instruments, Melville, NY, USA). Excitation was achieved with a Nikon Intenslight C-HGFI mercury lamp filtered with FitC and Texas Red filter sets. All single-labeled BrdU-positive cells were counted in the granule cell layer (GCL) and within two cell diameters below this region in the subgranular zone (SGZ). BrdU-positive cells were also counted in the dentate hilus, encompassed by the GCL and an imaginary border between the lateral endings of the GCL. Every 10th slice was collected for analysis along the dorsal hippocampus. The number of labeled cells was calculated in 8 coronal sections from each rat and expressed as the mean number of cells per section, then multiplied by 10.

### 4.3. Immunohistochemical Assay for Neurogenesis in the Dorsal Hippocampus

A quantitative neurogenesis assay was developed using colocalization of the fluorescent signal arising from BrdU and NeuN using confocal microscopy (Figure 3). The number of BrdU and NeuN double-labeled cells was quantified using a confocal scanning microscope (Leica DMI 6000B, Leica Microsystems, Deerfield, IL, USA). Excitation was achieved with a 488 nm Kr/Ar laser and a 594 nm He/Ne laser. Two separate photo multiplier tubes were used to scan for emissions at wavelengths 500 to 580 nm and 605 to 700 nm for the emissions from Alexa 488 and Alexa 594 fluorescent antibodies, respectively. Each of the eight sections collected per rat was examined for BrdU/NeuN colocalization rates. All BrdU-positive cells within one 246 μm^2^ field of view were examined for colocalized fluorescence with NeuN using a software colocalization tool (Leica LAS Advanced Fluorescence suite v.4, Leica Microsystems, Deerfield, IL, USA) which identifies and plots pixels with colocalized emission of different wavelengths above a specified intensity (Figure 3). The percentage of BrdU-positive cells expressing the NeuN neuronal marker was calculated as BrdU/NeuN-positive cells divided by the total number of BrdU-positive cells.

### 4.4. Statistical Analyses

Analysis of all experiments comparing control and drug-treated groups was performed using an unpaired *t*-test (one-tailed); *p* values  ≤  0.05 were considered significant. We used *t*-tests because the experiments were performed separately and had only two groups, control and drug-treated groups. Mitogenic, survival, and colocalization data were analyzed using an unpaired *t*-test (one-tailed), since rats were not paired and a prediction was made on which means would be greater based on studies with adult rats. Daily weights were compared using an analysis of variance (ANOVA) for repeated measures.

## 5. Conclusions

Previous observations have shown that drugs that inhibit serotonin reuptake, but not norepinephrine reuptake, have antidepressant-like effects on juvenile rats [8,24]. Furthermore, drugs that inhibit serotonin reuptake, but not norepinephrine reuptake, increase hippocampal expression of BDNF and its receptor TrkB in juvenile rats [9,10,20]. This study has found that antidepressant drugs do not alter the birth of newborn neurons or neurogenesis in pre-adolescent rats. However, in adolescent rats, an SSRI, but not a selective noradrenergic reuptake inhibitor, increases both the birth of newborn cells and adult neurogenesis in the hippocampus. It is indeed striking that this is well-correlated with the efficacy observed in the clinical response to antidepressant drug classes in adolescent humans. Taken together, these correlations suggest a role for BDNF and neurogenesis in the mechanism of action of SSRIs, but not noradrenergic reuptake inhibitors, in antidepressant drug action in adolescent humans. Moreover, these correlations suggest that BDNF and neurogenesis are involved in the observed developmental difference in antidepressant drug action in patients.

## Abbreviations

SSRI	Serotonin Selective Reuptake Inhibitor
BDNF	Brain-derived Neurotrophic Factor
TrkB	Tropomyosin Receptor Kinase B
BrdU	Bromodeoxyuridine
TCP	Tranylcypromine
